# Effectiveness of web application as educational media in increasing the caries risk knowledge and decreasing the caries risk score among dental students in Indonesia

**DOI:** 10.1186/s12903-021-01995-1

**Published:** 2021-12-15

**Authors:** Risqa Rina Darwita, Febriana Setiawati, Ishlah Fakhirah Rahmah

**Affiliations:** 1 Department of Dental Public Health and Preventive Dentistry, Faculty of Dentistry, Universitas Indonesia, Jalan Salemba Raya No. 4 Central Jakarta, Jakarta, Indonesia; 2 Undergraduate Student of Dental Public Health and Preventive Dentistry, Faculty of Dentistry, Universitas Indonesia, Jakarta, Indonesia

**Keywords:** Web application, Caries risk, Health promotion, Knowledge, Health practice

## Abstract

**Background:**

This study evaluating the effect of web application media in increasing the caries risk knowledge and decreasing the caries risk scores among dental students.

**Methods:**

A quasi-experimental design along with a purposive sampling technique was used in this study. A total of 361 undergraduate pre-clinical dental students from 15 universities in Indonesia were divided into two groups: intervention (n = 282) and control (n = 79). The students in the intervention group received a web application media with educational materials to independently check their caries risk, whereas those in the control group received the application without any educational materials. The students were instructed to use the web application at least once a week for 21 days and complete the pretest and posttest questionnaires and web application evaluation questionnaires. In addition, they were required to independently examine their initial and final caries risk.

**Results:**

A significant increase in the level of knowledge was observed in the intervention group, but not in the control group, after the use of the web application. Each group showed a decrease in the caries risk score, but the difference was not statistically significant before and after the use of a web application in both groups.

**Conclusion:**

These findings indicate that health promotion and education about caries risk through web application media can improve the knowledge and reduce the caries risk in dental dentistry students.

**Supplementary Information:**

The online version contains supplementary material available at 10.1186/s12903-021-01995-1.

## Background

Dental caries is a biofilm-mediated, diet-modulated, multifactorial, non-communicable, dynamic disease that results in net mineral loss of dental hard tissues [1]. Caries risk is the probability that caries lesions will appear or progress if conditions remain the same within a given period of time [1]. The dental caries risk is determined by biological, behavioral, psychosocial, and environmental factors, commonly known as caries risk factors [1, 2]. According to the Indonesian government’s 2018 basic health surveillance results, the prevalence of dental caries in Indonesia is 75.3%; the average DMF-T index among 15- to 24-year-olds (college students, in general) is 3.1, which lies within the moderate DMF-T category according to the World Health Organization [3, 4].

The strategies employed to reduce the prevalence of dental caries and caries risk include caries risk assessment (CRA) and health promotion with regard to the risk of dental caries and its factors [5–7]. A CRA is performed so that preventive measures can be used among people who are at high risk of dental caries. Health promotion allows individuals to be able to control the determinants or risk factors of dental caries in order to reduce the caries risk and, ultimately, reduce the prevalence of dental caries [5, 8]. This can be performed using media teaching aids/props to convey and demonstrate information during the educational or teaching process, thus making it easier to understand and more attractive to the student [9, 10].

Dental students show improved knowledge of dental caries from the first year until graduation, however, this learning process does not motivate them to practice the oral health self-care that they have learned. Dental students learn about oral health in college either directly from lecturers or independently by using educational media, such as Youtube, video, internet, etc. One of the effective ways to reduce dental caries disease in dental student is to practice oral health self-care, and dental student can measure the risk of dental caries independently [11, 12]. Recently, online learning using educational media is increasingly being incorporated into the medical and dental teaching methods due to the pandemic Covid-19 [13, 14]. With the development of information technology in the modern era, information about oral health such as dental caries risk should not be difficult to obtain. Web applications can play a role in improving oral health via the sharing of educational materials, which could motivate dental students to change their oral health behaviours in a way that decreases the dental caries risk [15, 16]. College students are one of the groups in society who mostly use the Internet as a source for learning activities. Web media, which present data through audio, visual, and audio–visual means, can increase student's interest and are proven to be more effective for sharing information [17–19]. According to a survey conducted by the Association of Indonesian Internet Network Providers, a total of 132.7 million Indonesians were connected to the Internet in 2016 [20].

Teaching media through web application can be used to increase knowledge and skills of oral health selfcare and preventive care of dental student to measure dental caries risk and its risk factors, which will increase the awareness of dental student of how to decrease the dental caries risk [8]. Thus, the aim of this study is to assess whether an educational media using web application about dental caries risk and its factors can improved dental student knowledge and skill to measure dental caries risk score among undergraduate pre-clinical dental students.

## Methods

### Design

A quasi-experimental with pre-test and post-test design study was conducted. The purposive sampling technique was used to select a dental student as a respondent from 15 universities in Indonesia.

### Ethical considerations

Participation in the study was voluntary, and the students completed and signed informed consent forms prior to participation. The study was approved by the Ethics Committee of the Faculty of Dentistry, Universitas Indonesia, No. 16/*Ethical Approval/FKGUI/VIII/2020* with No. Protocol 010160720.

### Participants

The undergraduate pre-clinical of the First-, Second-, and Third- grade dental students from 15 universities in Indonesia were invited to take part in the study, which was conducted from July 2020 to October 2020. The inclusion criterion for this study included to the first-grade students are students who enrolled faculty of dentistry in September 2020, and have never received a dental caries lecture, the second-grade students are students who have just gotten the basics of dental caries, the third year students are students who have received lectures on the risk of dental caries. The dental students aged 18–22 years who possessed a gadget with access to the Internet and web applications and were willing to participate in the study.

### Sample size

The sample size was calculated as 23 in each group with a study of power 99%, alpha of 5%, standard deviation of 2.09, and effect size of 2 [24]. This number was reached after adding 10% of the total sample size required, in case some of the students dropped out of the study.

Cluster probability proportional to size sampling was used in the sampling method, where the Faculty of Dentistry was the cluster. The total number of Faculty of Dentistry in Indonesia is 32, consisting of 25 in West Indonesia, 7 in Central Indonesia, 0 in East Indonesia. The total number of first to third- grade dental students in West Indonesia was 330 students, while the Central Indonesia cluster included 92 students.

Next, the sample size selection was adjusted based on the proportion of the number of students in each cluster of Faculty of Dentistry. Sampling was conducted randomly from the Faculties of Dentistry, resulting in nine Faculty of Dentistry which was used as the study sample was carried out randomly, the result was is 9 cluster faculties of dentistry from West Indonesia consisting of Sumatra, Kalimantan, Java, Bali and including 330 dental students (of which 282 were willing to participate), and 3 Faculties of Dentistry in Central Indonesia, consisting of Sulawesi only and including 92 dental students (of which 79 were willing to participate). Dental students from the West Indonesia cluster were heterogenous, whereas dental students from Central Indonesia cluster were almost homogenous. Thus, dental student from Central Indonesia were selected as the control group and dental students from West Indonesia were selected as the intervention group.

### Web application design

The web application media have been used to promote educational material, including to dental caries risk and its factors, how to assess the individual dental caries risk score independently. It was designed, adopted, and modified from Busby (2013), whereas the assessment method of caries risk is based on the Traffic Light Matrix (TLM) [16, 21]. The web application media contain educational material about caries risk and how it can be measured independently, based on the TLM.

In the present study, dental caries risk was examined using a web application media in the form of a questionnaire adopted and modified from the TLM method. This was accompanied by explanations and pictures to make it easier to carry out the examination independently. The respondents received the results at the end of the examination, which were categorized as low, moderate, or high risk based on the condition, behavior, and habits related to the caries risk factors.

### Intervention and instruments

Before the research begins, research preparation is carried out using the zoom link, the researcher explained to dental students about the research to be conducted in 21 days, all dental student will fill out the questionnaire as pre-test, to assess the dental caries risk is based on the Traffic Light Matrix (TLM) and in the last of study in the day 21th. The post-test and assessing the dental caries risk were performed.

**Fig. 1:**
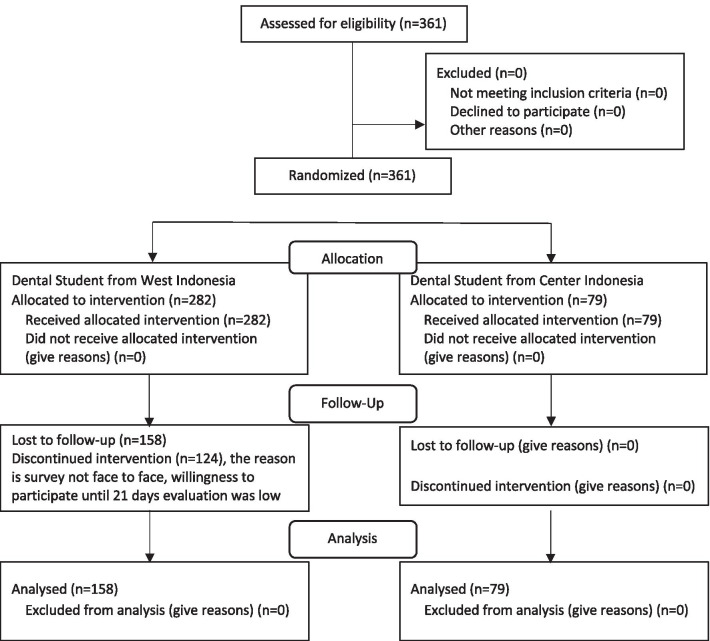
CONSORT flow diagram of the randomized controlled trial

The students were divided into two groups as follows (Fig. 1): Intervention group, comprising students who used the web application and power point slide along with educational materials (n = 282) including to the cause of dental caries, dental caries risk and its factors, and the skills of dental student how to assess the individual dental caries risk score independently.

Control group comprising students who used the web application contain educational materials (n = 79) in general about the cause of dental caries.

### Data collection

The students in both groups filled out a questionnaire (Additional file 1: Effectiveness of Web Application as Educational Media in Increasing the Caries Risk Knowledge and Decreasing the Caries Risk Score among Dental Students in Indonesia) after signing on to a web application. The questionnaire distributed to the dental student was G-form contains the following questions consist of the demographic information of participants including age, sex, grade, and university origin of dental student. Next, the 10 question about dental caries, dental caries risk and its factors which were designed to evaluate the knowledge of dental student. The 10 questions about the skill of dental student to assess the caries risk score by themself. The questionnaire was adapted and modified from previously available valid questionnaires [21–23]. The Content Validity Indexes showed that all items in the questionnaire were valid.

Students in intervention group were instructed to use the web application at least once a week for 21 days to read the educational materials and assess their dental caries risk score based on the Traffic Light Matrix (TLM) by themselves. After 21 days, both of intervention and control group of the students were instructed to complete the answer in web application evaluation questionnaires.

### Statistical analysis

Descriptive statistics, including the percentage, mean, and standard deviation, were reported. The answers to questions on knowledge were dichotomized as correct and incorrect. Each correct answer was scored as 1, and incorrect answers were scored as 0. The maximum and minimum scores were 10 and 0, respectively. The minimum and maximum scores that could be acquired for the questions on caries risk were 10 and 30, respectively, and were categorized as low (10–15), moderate (16–20), or high (20–30). Paired T-test was used to compare the pretest and posttest scores. Spearman's correlation test was used to determine correlations between the frequency of accessing the web applications and both caries risk knowledge and caries risk score.

## Results

Table 1 present the data of 361 students, mean age, 19 years, consist of boys, 16.6%; girls, 83.4% were analysed in this study. Only 158 students completed the whole study, resulting in a response rate of 43.8%. Of the 361 students, 282 belonged to the intervention group and 79 to the control group. A total of 203 students in the intervention group did not complete the final Caries Risk Score Questionnaire (posttest); nonetheless, their data were used to analyze the initial and final caries risk knowledge and the initial caries risk score The majority of the students in the intervention group were in the grade 1st, whereas those in the control group were in the grade 3rd. The differences of dental caries risk knowledge between grade 1st, grade 2nd and grade 3rd were analyze by Oneway Anova, the result shows a significant different of their knowledge on dental caries risk between grade 1st, grade 2nd and grade 3rd (*p* < 0.001). Overall, 63.4% and 36.6% of the students were from universities inside and outside the Java region, respectively.

**Table 1:** Demographic information of dentistry students in intervention and control groups

Variable	Category	Intervention groups		Control groups		*p* value
		Number	%	Number	%	
Gender	Girls	234	83	67	84.8	0.586
	Boys	48	17	12	15.2	
Level of study	Grade 1st	105	37.2	9	11.4	0.0001
	Grade 2nd	89	31.6	48	60.8	
	Grade 3rd	88	31.2	22	7.8	
Universities (region)	Java	182	64.5	47	59.5	
	Outside Java	100	35.5	32	40.5	
Age	Average	19.1		19.3		
	Standard deviation	1.1		0.7		

### Knowledge score before and after the intervention

The maximum total knowledge score that could be acquired was 10. The differences between score knowledge of intervention group compared to control group were analyze by T-test. The result after intervention 21 days shows that the mean and standard deviation of total knowledge scores acquired by the intervention and control groups in the pretest were 7.98 ± 1.45 and 8.13 ± 1.29, respectively, which increased to 9.39 ± 1.042 and 8.54 ± 1.26, respectively. A significant difference (*p* < 0.01) was found in the comparison between the intervention group and control groups for the knowledge gap, as shown in Table 2, before and after using web application.

**Table 2:** The comparison of before and after score gap of knowledge using application web in intervention and control groups

Group	Score gap		*p* value
	Average	Standard deviation	
Intervention group	1.14	0.41	< 0.01
Control group	0.41	0.03	

### Caries risk score before and after the intervention

The majority of the dental students were in the moderate caries risk category with a mean total score of 16. The minimum and maximum total caries risk scores that could be acquired was 10 and 30, respectively. The mean total caries risk score for the pretest was 16.47 and 16.54 in the intervention and control groups, respectively. These values decreased to 15.71 and 15.81 in the intervention and control groups, respectively (Table 3). Statistically significant differences (*p* < 0.05) in the caries risk score were observed before and after using the web application in both groups.

**Table 3:** The mean total caries risk score acquired before/after using application web in intervention and control groups

Group	Before the intervention		After the intervention		*p* value
	Average	Standard deviation	Average	Standard deviation	
Intervention group	16.47	2.02	15.71	2.29	< 0.01
Control group	16.54	1.89	15.81	1.97	< 0.01

### Correlation between frequency of accessing the web applications and both caries risk score and caries risk knowledge score

**Fig. 2:**
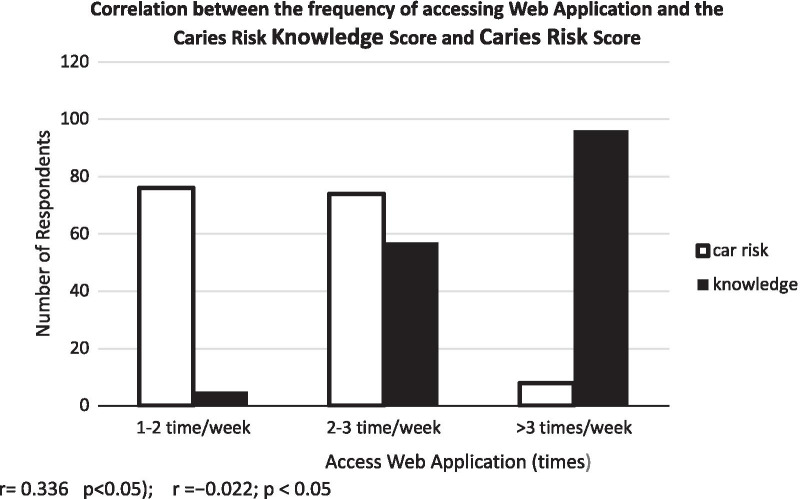
Correlation between the frequency of accessing web application and the caries risk knowledge score and caries risk score

Figure 2 shows the correlation between the frequency of accessing the web application and both of knowledge score about caries risk, and assessing of caries risk score was acquired using Spearman's correlation test.

A significant relationship (r = 0.336; *p* < 0.05), between the frequency of accessing the web application and caries risk knowledge score. Likewise, a significantly weak negative linear relationship (r =  − 0.022; *p* < 0.05) was observed between the frequency of accessing a web application and caries risk score.

## Discussion

This study assessed the effectiveness of web application media in increasing the caries risk knowledge and decreasing the caries risk score among dental students from July to October 2020, which coincided with the admission of new students. The results showed an improvement in caries risk knowledge and reduction in caries risk score after using the application. However, only the group who were given educational materials in addition to the web application (intervention group) showed a significant increase in caries risk knowledge. Aside from the background of the study, the respondent was an undergraduate pre-clinical dental students from the grade 1st, grade 2nd and grade 3rd of education, whereas learning process does not motivate them to practice the oral health self-care that they have learned. Dental students learn about oral health in college either directly from lecturers or independently by using educational media [11, 12]. Providing intervention with web application media and additional dental caries risk material using power point slide to the intervention group, shows that the message conveyed will be more interesting to increase attention in the learning process, because with high attention it will increase the motivation of the intervention group students to learn better, however the additional dental caries risk material using power point slide will be more recorded that important in learning process so that students will remember it better, Diah et al. (2021) state that most adolescents used Google, social media, website to seek oral health information to expand and develop their creativity for searching health information. [24, 25]. In addition, by getting messages through the web application and dental caries risk materials using power point slide once a week can communicate messages quickly to increase oral health knowledge which has an impact on students' understanding of dental caries risk faster which impact to oral health literacy, increase attitude and behaviour of dental student in intervention group compared to control group that only get web application, whereas control group only get the information from web applications that must be viewed at least once in 21 days of study. So the results of this study can be said that the use of web application media and power point slide media is more effective than only using web application media which is given to the control group. The results of this study are in line with McGrath's (2019) statement, namely the more often someone gets oral health promotion that supports behaviour change the tend to be more successful [26].

In the present study, the mean total pretest and posttest scores were significantly improved in the intervention group and only slightly increased in the control group after using the web application for 21 days. A statistically significant difference in caries risk knowledge was noted before and after using the web application in the intervention group, but not in the control group. This might be because, unlike the intervention group, the control group only used the web application and did not receive any educational materials through power point slide. According to Edgar Dale's theory in Ken Master (2020), the intervention group received information through what they saw or read and interpreted it as knowledge. The more the senses are involved in an educational process, the easier it will be for the educational target to accept and remember [27]. In addition, the use of educational media that are interesting and easy to understand can significantly boost the increase in knowledge [28]. The educational material that the respondents were given about dental caries risk through Power Point presentation could also be easily found through Google search. However, for students to implement the educational material regarding caries risk in their everyday lives, it is necessary to provide all the information at once in an easily accessible platform such as a web application (accessible from their smartphone, tablet, laptop, computer, etc.).

The dental students are privileged to and exposed to dental education apart from the intervention. Based on the curriculum, they will be exposed to it since the beginning of the study. The dental education system in Indonesia accepts candidates from various socioeconomic backgrounds who become eligible to study in faculty of dentistry based on their score in state entrance examinations. Lisa et al. (2020) reported in their study that the dental curriculum for undergraduate student in Indonesia comprises to 4 years, consists to Preclinical student from the years 1–3, while the clinical student is perform in the years 4–5. During a pandemic covid -19 from 2020 until now the faculty of dentistry in Indonesia using a blended learning, the blended learning provides better student’s satisfaction, motivation, student engagement and performance. In line with the learning system implemented by dentistry students in Indonesia, it can support research results using web applications more effectively to increase knowledge and motivation of dental students in reducing the risk of dental caries [29]. Dena [30] elucidated that dental students acquire knowledge regarding oral healthcare maintenance and disease prevention within the various dental courses as they progress in their dental training. The findings of this study provides a synthesis of a web application media as a digital media which use at least once a week for 21 days in intervention group of dental student can give a benefit, including audience engagement and information retention to increasing their knowledge and motivating the attitude of dental student to improvement in their oral health behaviour as well as elucidating by Mc Grath [26] about the common behavioural models and theory of planned behaviour and the health believe model, that is, the more often the intervention group of dental student gets advocacy and health promotion from web-application media, their knowledge will increase and effect the attitude and oral health behaviour that support to decrease the dental caries risk score, compared to the control group who got knowledge about the risk of dental caries only from learning at the faculty.

The mean total caries risk score in the intervention and control groups decreased after the use of the web application media in this study, indicating that there was a change in the oral health behavior of the dental students after using the web application for 21 days. This finding was in accordance with the statement by Aljafari et al. [25], who suggested that caries risk was decreased after a behavior change was made by implementing suggestions for the prevention or improvement of caries risk factors. In the present study, the web application was used not only as educational media but also as media/tool to examine the caries risk. This allowed students to be able to assess their caries risk independently, making them aware of the factors that were assessed, the caries risk factors present, and the caries risk category they belonged to. The perception of susceptibility to dental caries is developed when the Caries Risk Assessment is carried out and the results are shown. The health belief model theory suggests that a person will perceive those conditions as a vulnerability to risk factors, namely, the risk of dental caries [27, 28, 31]. This situation might have motivated the students to improve their oral health behavior based on their respective risk factors, and eventually, the outcome of a decreased caries risk score was achieved. This finding was in alignment with the study conducted by Schmidt et al. [32], which stated that the application of the health belief model in oral health education can improve the behavior of maintaining oral health.

The test results in the present study showed that there was a significant weak positive linear relationship between the frequency of accessing the web application and caries risk knowledge. Alternatively, a significantly weak negative linear relationship was detected between the frequency of accessing the web application and caries risk score. Thus, the more often the students accessed the web application, the higher the caries risk knowledge score and the lower the caries risk score. This was in accordance with the findings of another study that showed that the more often someone read or repeated the information provided, the higher the increase in their understanding [9, 31]. However, the weak negative linear relationship between the frequency of accessing the web application and the caries risk score in this study might be because the decrease in caries risk was influenced not only by the individual's understanding about something but also by other factors such as their attitudes and behavioral practices [25, 32].

There are several limitations to this study. The researcher could not control the presence of other interventions in the student's environment, such as their schedules, which could affect their interest in reading the educational materials or changes in behaviors related to the caries risk factors. The TLM CRA was used to assess the caries risk. This test was modified to facilitate the independent assessment of caries risk by respondents, which might have affected the sensitivity of the test. In addition, the interventions in the form of web applications as educational media were highly dependent on the willingness of each respondent. The researcher could not provide routine interventions every week. The knowledge scores might have been higher and caries risk scores lower if the interventions were carried out more routinely.

## Conclusions

The results of the study prove that the more often of dental student uses the web application indicates the significantly increases in knowledge score of dental caries risk and its factors, and significantly decreases dental caries risk score.

The findings of this study indicates that web applications can support dental student lectures in increasing knowledge and skills to measure caries risk themselves among dental students, so that it will have an impact on increasing awareness of oral health behaviour among dental student. In addition, the result of this study show that it is more effective if the web applications is added with dental caries risk material using power point slide.

Therefore, it can be concluded that the web application is effectively used to support learning in order to improve the knowledge and skills to measure dental caries risk score among undergraduate pre-clinical dental students.

## Supplementary Information

**Additional file 1:** The main Data of answer of questionnaire of all respondents about result of evaluation of dental caries risk and the knowledge of respondent after using application web media.

## Data Availability

Additional file 1. Questionnaire Effectiveness of Web Application as Educational Media in Increasing the Caries Risk Knowledge and Decreasing the Caries Risk Score among Dental Students in Indonesia. Risqa Rina Darwita, Febriana Setiawati, Ishlah Fakhirah Rahmah. The contains of questionnaire following about demographic data, caries risk knowledge, caries risk score, and web application evaluation in Excel format.

## Abbreviations

CRA: Caries risk assessment
TLM: Traffic light matrix
